# Multidisciplinary Management of Emergency Neurosurgery for Intracerebral Hemorrhage During Pregnancy: A Case Report

**DOI:** 10.3390/healthcare14111534

**Published:** 2026-06-01

**Authors:** Eleonora Case, Sabrina Bettoni, Rossana Maria Mosca, Fabio Mauri, Vladimir Reyes Lozano, Rafaela Garrido, Paolo Maino, Alexandros Moniakis, Davide Milani

**Affiliations:** 1 Department of Nursing, Ente Ospedaliero Cantonale (EOC), Regional Hospital of Lugano, 6900 Lugano, Switzerland; rafaela.garrido@eoc.ch; 2 Department of Anesthesiology, Intensive Care and Emergency Medicine, Ente Ospedaliero Cantonale (EOC), Regional Hospital of Lugano, 6900 Lugano, Switzerland; sabrina.bettoni@eoc.ch (S.B.); vladimir.reyeslozano@eoc.ch (V.R.L.); paolo.maino@eoc.ch (P.M.); 3 Department of Gynecology and Obstetrics, Ente Ospedaliero Cantonale (EOC), Regional Hospital of Lugano, 6900 Lugano, Switzerland; rossanamaria.mosca@eoc.ch (R.M.M.); fabio.mauri@eoc.ch (F.M.); 4 Faculty of Biomedical Sciences, Università della Svizzera Italiana, 6900 Lugano, Switzerland; 5 Institute of Clinical Neurosciences of Southern Switzerland, Department of Neurosurgery, Ente Ospedaliero Cantonale (EOC), Regional Hospital of Lugano, 6900 Lugano, Switzerland; alexandros.moniakis@eoc.ch (A.M.); davide.milani@eoc.ch (D.M.)

**Keywords:** intracerebral hemorrhage, pregnancy, craniotomy, multidisciplinary management, fetal monitoring, neurosurgical emergency

## Abstract

**Background:** Acute neurosurgical emergencies during pregnancy are rare but pose significant challenges due to the need for simultaneous management of two interdependent patients. Evidence remains limited, and standardized multidisciplinary protocols are lacking. **Case Presentation:** A 38-year-old woman at 32 + 4 weeks of gestation presented with acute left hemiparesis secondary to right capsulo-insular intracerebral hemorrhage with mass effect. Following initial conservative management, neurological deterioration and hematoma expansion necessitated emergency craniotomy. A structured multidisciplinary approach was implemented involving neurosurgery, anesthesiology, obstetrics, and neonatology, with predefined roles, continuous intraoperative fetal monitoring, and readiness for emergency cesarean delivery. Anesthetic management balanced maternal neuroprotection with preservation of uteroplacental perfusion. Surgery was completed without fetal compromise. The patient demonstrated neurological improvement and was transferred on postoperative day 13. Elective cesarean delivery was performed at 36 weeks. **Conclusions:** This case illustrates that emergency neurosurgery during pregnancy may be feasible in selected settings when supported by structured multidisciplinary coordination. Key practical elements included continuous fetal monitoring, predefined team roles, and immediate availability of obstetric and neonatal support. In this individual case, these components facilitated intraoperative decision-making and were associated with favorable maternal and fetal outcomes; however, their individual contribution cannot be determined from a single clinical experience. Further evidence is needed to assess the applicability of this approach across different clinical scenarios.

## 1. Introduction

Emergency neurosurgery typically focuses on a single patient. However, in pregnant women beyond fetal viability, intracerebral hemorrhage represents a dual-patient scenario requiring explicit prioritization of maternal survival while simultaneously addressing fetal well-being [1,2,3]. Although acute neurosurgical events during pregnancy are rare [3,4], their complexity arises from the need for simultaneous, coordinated care of two interdependent patients, necessitating collaboration among specialties that often function independently, including neurosurgery, anesthesia, obstetrics, and neonatology [3,5,6].

Intracerebral hemorrhage (ICH) during pregnancy is rare but carries substantial morbidity and mortality [4,7]. The incidence of intracranial bleeding during pregnancy is approximately 12.2 per 100,000 pregnancies, with hemorrhagic stroke occurring in 20–40 per 100,000 pregnancies overall [3,7]. Unlike the general population, in which approximately 85% of strokes are ischemic, pregnancy-associated stroke has a substantially higher proportion of hemorrhagic events, accounting for nearly half of all cases [3]. The risk is highest during the third trimester and the early postpartum period [7].

Maternal mortality rate for pregnancy-associated intracerebral hemorrhage can reach 20% [3]. Fetal outcomes are similarly concerning, with fetal death occurring in 3.4% of cases—a five-fold increase compared to pregnancies without intracerebral hemorrhage (OR 5.40, 95% CI 2.67–10.92) [8]. These data highlight that pregnancy-associated intracerebral hemorrhage remains a severe obstetric emergency, carrying a high risk of maternal and fetal morbidity and mortality [2,3,8].

Intracerebral hemorrhage in pregnancy requires systematic consideration of both primary and secondary causes [3,9]. Primary hemorrhage is most commonly associated with hypertensive disorders of pregnancy (preeclampsia, eclampsia, HELLP syndrome), which disrupt cerebral autoregulation and the blood–brain barrier. Secondary causes include rupture of cerebrovascular malformations as well as less frequent conditions such as cerebral venous sinus thrombosis, reversible cerebral vasoconstriction syndrome, coagulopathies, and hemorrhagic transformation of ischemic stroke or intracranial neoplasms [3,4,9,10]. Prompt diagnostic assessment with neuroimaging (CT, CT angiography, and MRI/MR angiography) is essential to identify the underlying etiology and guide management and should not be withheld because of pregnancy [3,11].

Current stroke prevention and treatment guidelines offer limited guidance for managing stroke in pregnant and postpartum patients, largely due to the exclusion of pregnant individuals from major stroke trials [3,7]. The Canadian Stroke Best Practice Consensus Statement (2018) was the first to specifically address this gap, recommending that intracerebral hemorrhage during pregnancy be managed similarly to nonpregnant patients, with maternal safety as the primary goal and coordinated multidisciplinary care suggested [12]. More recently, the 2026 American Heart Association/American Stroke Association (AHA/ASA) Scientific Statement on Prevention and Treatment of Maternal Stroke reinforced these principles, emphasizing that pregnancy should not delay evidence-based diagnosis and treatment [3]. However, the existing literature mainly addresses maternal neurosurgical management and fetal or obstetric monitoring separately, with limited practical guidance on real-time coordination among neurosurgical, anesthetic, obstetric, and neonatal teams during acute emergencies [2,3,13,14].

This case report describes the management of a pregnant woman undergoing emergency craniotomy for intracerebral hemorrhage during viable pregnancy, in the context of hemorrhagic metastatic melanoma. The novelty of this report lies in the integrated perioperative management strategy combining continuous intraoperative fetal monitoring, preparedness for emergency cesarean delivery, tailored anesthetic management, and real-time multidisciplinary coordination. As a carefully documented single case, this report is not intended to establish the safety or generalizability of a specific protocol. Rather, it aims to share multidisciplinary lessons from a rare and high-risk maternal–fetal neurosurgical emergency. By detailing the operational coordination among neurosurgery, anesthesia, obstetrics, and neonatology, this report aims to offer practical insights relevant to perioperative planning and multidisciplinary discussion in similarly complex emergency settings.

### Reporting Standards

This case report was prepared in accordance with the CARE (CAse REport) guidelines for transparent reporting of case reports. Written informed consent for publication of the case details and accompanying images was obtained from the patient.

## 2. Case Presentation

### 2.1. Patient Information and Chief Complaint

A 38-year-old pregnant woman (gravida 2, para 1) at 32 weeks and 4 days of gestation presented to the emergency department with acute onset of left-sided hemiparesis.

The patient was last known well at 11:30 and subsequently developed mild left-hand weakness, initially attributed by the patient to fatigue. At 12:30 she experienced sudden worsening with rapid progression to complete left-sided hemiparesis. She arrived at the emergency department at 13:15, approximately 105 min after symptom onset.

Her obstetric history included one previous uncomplicated term vaginal delivery. The current pregnancy had been uncomplicated, with normal fetal growth and no obstetric concerns at routine prenatal visits. No history of hypertensive disorders or gestational diabetes were reported. Past medical history was notable for well-controlled bronchial asthma treated with inhaled salbutamol as needed and a previously excised dorsal cutaneous melanoma (2018; stage not known). She had no history of hypertension, diabetes, thrombophilia, coagulopathy, or prior cerebrovascular events.

Current medications included prenatal vitamins and oral iron supplementation. No anticoagulant or antiplatelet therapy was ongoing, and no drug allergies were reported. No family history of cerebrovascular disease, coagulopathy, or malignancy was reported. The patient was a non-smoker with no alcohol or recreational drug use.

### 2.2. Clinical Findings

On admission, vital signs were stable (blood pressure 115/72 mmHg, heart rate 80 bpm, oxygen saturation 97% on room air, respiratory rate 16 breaths/min, temperature 36.7 °C). General physical examination was unremarkable.

Neurological examination revealed severe left facio-brachio-crural hemiparesis (Medical Research Council grade 0/5), left homonymous hemianopia, and mild dysarthria. Glasgow Coma Scale score was 15, and the National Institutes of Health Stroke Scale (NIHSS) score was 18. Mild headache was present without photophobia, altered mental status or signs of meningismus. No seizure activity, vomiting or recent trauma were present. Because of the pregnancy status and the acute neurological presentation, preeclampsia/eclampsia-related neurological complications were specifically considered. However, no clinical features suggestive of hypertensive disorders of pregnancy were observed, including severe headache, visual scotomata, epigastric pain, hyperreflexia, or clonus.

Serial non-invasive blood pressure monitoring was initiated in the emergency department. Blood pressure remained stable throughout the initial observation period, with systolic values ranging from 115 to 130 mmHg and diastolic values from 70 to 75 mmHg. Antihypertensive therapy was therefore not required. Following ICU admission, invasive arterial monitoring was used to guide hemodynamic management, with the goal of avoiding hypotension and maintaining adequate uteroplacental perfusion.

Obstetric evaluation confirmed maternal–fetal stability. Findings were consistent with gestational age, with reassuring fetal status demonstrated by a fetal heart rate of 140–150 beats/min and a reassuring cardiotocographic pattern. No uterine contractions, vaginal bleeding, rupture of membranes, or evidence of active labor were observed.

### 2.3. Timeline of Clinical Events

The key clinical events from symptom onset through diagnostic evaluation, multidisciplinary decision-making, surgical treatment, and postoperative follow-up are summarized in Table 1. The timeline highlights the critical transition from initial conservative management to emergency surgical intervention, which was prompted by neurological deterioration and radiological evidence of hematoma expansion.

### 2.4. Diagnostic Assessment

Urgent non-contrast head CT (recorded dose-length product DLP 459 mGy·cm) was performed and demonstrated a heterogeneous right capsulo-insular hemorrhage measuring 50 mm × 40 mm × 35 mm with an estimated volume of approximately 35 mL (calculated using the ABC/2 ellipsoid formula). Imaging demonstrated a mass effect with 4 mm midline shift in the septum pellucidum and compression of the ipsilateral lateral ventricle, without radiological signs of transtentorial or uncal herniation.

Urgent brain MRI with MR angiography was performed to further characterize the lesion. MRI confirmed a right insular intra-axial hematoma (50 mm × 38 mm × 35 mm) with an estimated volume of approximately 33 mL with surrounding edema, mass effect, and a 4 mm midline shift in the septum pellucidum. No aneurysms or additional vascular malformations were identified. Imaging could not exclude an underlying lesion, including cavernous malformation or hemorrhagic metastasis.

Laboratory investigations, including complete blood count, coagulation profile, liver enzymes, renal function, and urine protein/creatinine ratio were unremarkable except for mild anemia (Hb 110 g/L) excluding pregnancy-specific neurological emergencies (preeclampsia, eclampsia, and HELLP syndrome) and coagulopathies.

Obstetric evaluation with ultrasound confirmed a viable female fetus with appropriate growth, reassuring fetal heart rate (140 bpm), and no signs of labor.

The initial diagnosis was spontaneous intracerebral hemorrhage of uncertain etiology in a patient at 32 + 4 weeks of gestation. Differential diagnosis included hypertensive hemorrhage despite normotension, occult vascular malformation, pregnancy-related cerebrovascular disease, and metastatic disease. Given the patient’s history of cutaneous melanoma, together with the atypical location and radiological appearance of the hemorrhage, hemorrhagic metastatic melanoma was considered among the leading diagnostic possibilities from the outset. Definitive diagnosis of hemorrhagic metastatic melanoma was established by histopathological examination of the surgical specimen obtained during craniotomy and hematoma evacuation. Histopathological examination confirmed metastatic melanoma, demonstrating melanin-containing atypical cells with immunohistochemical positivity for SOX10 and Melan-A, together with BRAF V600E mutation.

### 2.5. Therapeutic Interventions

#### 2.5.1. Multidisciplinary Planning

Following informed consent, a structured multidisciplinary management plan was established involving neurosurgery, anesthesiology, obstetrics, and neonatology, with predefined roles to ensure coordinated dual-patient care.

#### 2.5.2. Anesthetic Management

Preoperative status: On operating room admission, the patient had Glasgow Coma Scale score 14, NIHSS 19, and was classified as ASA physical status IV-E (emergency).

- Induction and airway management: Following preoxygenation, a modified rapid sequence induction was performed in a head-up position with left lateral tilt. Anesthesia was induced with propofol (2 mg/kg), rocuronium (1.2 mg/kg) and remifentanil (1.5 μg/kg). No premedication was administered. Tracheal intubation was performed using video laryngoscopy with a tracheal tube with stylet.
- Monitoring: Standard ASA monitoring including continuous end-tidal CO_2_ plus invasive arterial blood pressure monitoring via radial arterial catheter.
- Anesthesia maintenance: Total intravenous anesthesia (TIVA) using target-controlled infusion (TCI) with propofol (target plasma concentration 3–4 mcg/mL) and remifentanil (target effect-site concentration 3–6 ng/mL).
- Ventilation: Volume-controlled ventilation was used with tidal volume 6–8 mL/kg ideal body weight, PEEP 0–5 cmH_2_O, and respiratory rate titrated to maintain normocapnia (PaCO_2_ 35–38 mmHg) and SpO_2_ > 95%. End-tidal CO_2_ (EtCO_2_) was monitored continuously and serial arterial blood gas analyses were performed to confirm PaCO_2_ targets.
- Hemodynamics: Hemodynamic stability was achieved with individualized anesthetic titration, balanced crystalloid administration and continuous low-dose peripheral intravenous norepinephrine infusion (0.03–0.05 μg/kg/min). Systolic blood pressure was targeted to 130–140 mmHg. Norepinephrine was initiated at anesthetic induction and discontinued shortly before complete emergence from anesthesia.
- Fluids: Balanced crystalloid solutions (Ringer’s acetate) were administered to maintain euvolemia and adequate uteroplacental perfusion. Osmotic therapy was not administered.
- Antibiotic prophylaxis: Cefuroxime 1.5 g IV was administered 30 min before skin incision.
- Antithrombotic prophylaxis: No pharmacological thromboprophylaxis was administered perioperatively given active intracerebral hemorrhage and neurosurgical intervention. Mechanical prophylaxis with intermittent pneumatic compression devices was used.
- Antiepileptic prophylaxis: No prophylactic antiseizure medication was administered. The patient had no clinical seizures preoperatively or intraoperatively.
- Betamethasone 12 mg intramuscularly was administered in two doses 24 h apart for fetal lung maturation (day 0 at 18:00 and day 1 at 18:00). No additional corticosteroids for vasogenic cerebral edema were given perioperatively.

#### 2.5.3. Obstetric Management

- Continuous fetal heart rate monitoring was incorporated into intraoperative decision-making alongside maternal physiological variables.
- Tocolysis: To minimize the risk of perioperative uterine contractions, continuous intravenous hexoprenaline infusion (6.4 µg/h) was commenced at the time of surgical decision-making and continued through postoperative day 1.
- Predefined criteria for emergency cesarean included bradycardia, persistent decelerations, sinusoidal pattern, and sustained tachycardia [17].
- Maternal positioning: Left lateral decubitus with head elevation optimizing uteroplacental perfusion, cerebral venous return, and intracranial pressure while minimizing aortocaval compression.
- Prepared, disinfected and fully equipped surgical abdominal field with obstetric personnel and instrumentation immediately available for rapid cesarean if indicated.
- Dedicated neonatology team present and ready for immediate neonatal resuscitation if required.

From anesthetic induction through the intraoperative phase, progressive reduction in fetal heart rate variability and reactivity was observed, consistent with the sedative effects of maternal anesthesia (Figure 1 and Figure 2) [18]. Nevertheless, baseline fetal heart rate (FHR) remained within the normal range (110–160 bpm) without significant fluctuations, and no uterine contractile activity was recorded. No decelerations or suspicious patterns occurred throughout intraoperative observation (Figure 2). During emergence from anesthesia, gradual recovery of fetal variability and reactivity was documented (Figure 3).

These changes were interpreted in the context of maternal anesthetic depth and hemodynamic stability, avoiding unnecessary intervention while maintaining close surveillance [18,19].

#### 2.5.4. Neurosurgical Procedure

The neurosurgical intervention was planned and conducted considering both the urgency of maternal neurological deterioration and the obstetric context. The indication for urgent surgery was based on progressive neurological decline (NIHSS 18 → NIHSS 19) and hematoma expansion (from ~33 mL to ~61 mL) with increasing mass effect, which outweighed the risks associated with ongoing pregnancy.

A right temporal craniotomy was performed for hematoma evacuation and lesion exploration, allowing mass effect control, intracranial pressure reduction, and tissue sampling for histopathology. Surgery was performed with staged hemostasis at each anatomical layer to minimize intraoperative blood pressure fluctuations.

Critical intraoperative phases—including dural opening, hematoma evacuation, and brain manipulation—were associated with potential intracranial pressure and hemodynamic variations. During these phases, communication among the neurosurgery, anesthesia, and obstetric teams was intensified to allow real-time assessment of maternal and fetal status.

Continuous cardiotocographic monitoring enabled correlation between surgical steps and fetal heart rate patterns confirming absence of fetal distress and allowing continuation of the procedure.

### 2.6. Follow-Up and Outcomes

#### 2.6.1. Immediate Postoperative Period

Following controlled emergence from anesthesia, the patient was cooperative with spontaneous respiration and stable hemodynamics. Cardiotocographic monitoring demonstrated reassuring fetal status with baseline fetal heart rate 150–160 bpm, normal variability, and reactive pattern. No decelerations were observed. Postoperative MRI confirmed successful hematoma evacuation with significant reduction of mass effect compared with preoperative imaging (Figure 4).

#### 2.6.2. Intensive Care Unit

The patient was admitted to the ICU for continuous monitoring for 48 h. Serial neurological assessments documented progressive improvement: GCS improved from 14 to 15 within 24 h. Left-sided motor strength improved from M0 (preoperative) to M3 (Medical Research Council scale) by postoperative day 2. Hemineglect showed progressive improvement and resolution. No adverse events occurred during the ICU stay, including no rebleeding, seizures, surgical site infection, thromboembolic events, or obstetric complications. Antithrombotic prophylaxis was provided with enoxaparin 40 mg administered subcutaneously once daily. No routine prophylactic antiepileptic treatment was administered. Intravenous clonazepam (1 mg) was kept available as rescue therapy in the event of seizure occurrence, in coordination with the obstetric team. The patient remained seizure-free throughout hospitalization. Daily cardiotocographic monitoring confirmed sustained fetal well-being.

#### 2.6.3. Neurosurgery Ward

On postoperative day 2, the patient was transferred to the neurosurgery ward with an NIHSS of 14. Progressive neurological improvement continued throughout the ward stay. Serial obstetric assessments, including cardiotocography and ultrasound, confirmed appropriate fetal growth and well-being. No maternal complications (rebleeding, seizures, infection, venous thromboembolism) were documented during this period. Histopathological analysis confirmed metastatic melanoma, consistent with the patient’s prior history of dorsal cutaneous melanoma. This finding established hemorrhagic brain metastasis as the cause of the intracerebral hemorrhage.

#### 2.6.4. Transfer and Delivery

On postoperative day 13, the patient was transferred, with an NIHSS of 5, to a tertiary referral center to facilitate family proximity and coordinated oncological, obstetric, and neonatal care.

Cross-border collaboration ensured continuity of the management plan, though its contribution to outcome cannot be independently assessed. Due to progressive maternal clinical deterioration related to the underlying oncological disease, an elective cesarean section was performed at 36 + 0 weeks of gestation (three weeks after craniotomy). The procedure was uncomplicated and the neonate demonstrated adequate adaptation to extra-uterine life. Detailed neonatal data, including birth weight, Apgar scores, and umbilical cord arterial pH, were not available to the authors, as delivery and neonatal care were managed at the receiving institution. After 48 h, the patient was transferred to the Oncology department to start targeted oncological care. Long-term maternal neurological and oncological outcomes were not available, as subsequent care was provided at the referral center. This represents a limitation of the present report.

### 2.7. Patient Perspective

A formal patient perspective could not be obtained due to the patient’s transfer to a tertiary center and her subsequent oncological decline. During the perioperative period, the patient and her family were counseled on the dual-patient management strategy, the risks of emergency imaging and surgery, and the rationale for fetal monitoring. Verbal consent was obtained for all interventions. This remains a limitation of the present report.

## 3. Discussion

This report describes the perioperative management of a pregnant patient at 32 weeks’ gestation who underwent emergency craniotomy for intracerebral hemorrhage, with a final diagnosis of hemorrhagic melanoma brain metastasis. As a single case report, it does not establish safety standards or reproducible protocols. Rather, it illustrates a patient-specific clinical pathway, time-sensitive evaluation, and multidisciplinary coordination that guided management in this individual patient and may offer practical lessons for clinicians encountering similar presentations.

### 3.1. Clinical Reasoning and Decision-Making

The acute neurological presentation required rapid exclusion of several time-sensitive diagnoses including ischemic and hemorrhagic stroke, hypertensive disorders of pregnancy, cerebral venous thrombosis, ruptured vascular malformations, coagulopathies and neoplastic lesions [3,4,9].

Unlike standard neurosurgical emergencies, management of intracerebral hemorrhage in pregnancy requires simultaneous consideration of two interdependent patients [1,12,20]. Maternal stabilization remains the priority as maternal deterioration directly impacts fetal prognosis [2,7,14,21]. However, fetal well-being must also be considered once viability has been achieved. [14,21].

This patient presented with acute focal neurological symptoms with a clinical timeline consistent with presentation within the 4.5 h reperfusion window for acute ischemic stroke [22] (last known well time 11:30 and emergency department arrival 13:15). Given the differential diagnosis of ischemic versus hemorrhagic stroke, urgent non-contrast head CT was performed to rapidly differentiate between pathologies and guide time-sensitive treatment decisions. In accordance with the American College of Obstetricians and Gynecologists (ACOG) and American Heart Association/American Stroke Association (AHA/ASA) recommendations, imaging was not delayed because of pregnancy, as the maternal benefit of prompt diagnosis enabling or excluding reperfusion therapy outweighed the negligible fetal risk [3,11].

A low-dose non-contrast head CT protocol was selected to minimize radiation exposure while maintaining diagnostic image quality, in accordance with the ALARA principle (the recorded dose-length product was 459 mGy·cm). Given the distance between the imaging field and the uterus, fetal radiation exposure was considered negligible [11]. Lead shielding of the gravid uterus was not applied, consistent with current evidence demonstrating negligible benefit from patient contact shielding during CT and potential for paradoxically increased scatter radiation [23,24]. The negligible fetal risk [11] and the clinical indication of immediate imaging were discussed with the patient, and verbal consent was obtained. Non-contrast head CT demonstrated a heterogeneous right capsulo-insular hemorrhage, excluding acute ischemic stroke and obviating the need for reperfusion therapy. Neurosurgical evaluation and blood pressure control were promptly initiated targeting systolic blood pressure to 130–140 mmHg as indicated in AHA/ASA 2022 guidelines and in the Code ICH consensus [10,15,25].

Following initial non-contrast CT findings, urgent brain MRI with MR angiography was performed to further characterize the lesion and assess for an underlying structural etiology such as vascular malformation, aneurysm, or neoplasm. MRI confirmed a right insular hematoma while excluding aneurysms or additional vascular malformations. Imaging could not exclude an underlying lesion, including cavernous malformation or hemorrhagic metastasis. Anticoagulant exposure, coagulation abnormalities, hypertensive disorders of pregnancy and venous thrombosis were also excluded, narrowing the differential diagnosis toward a neoplastic etiology.

Hemorrhagic melanoma brain metastasis was confirmed postoperatively by histopathological examination and significantly influenced subsequent management. Melanoma is among brain metastases most frequently associated with hemorrhage [26,27] and may clinically and radiologically mimic primary intracerebral hemorrhage. Pregnancy-associated melanoma complicated by brain metastases is rare and generally associated with poor prognosis [28,29]. In the present case, diagnosis prompted transfer to a tertiary referral center for integrated care (oncological staging and treatment, coordinated obstetric–oncological delivery planning). Given the known risk of placental metastasis in melanoma [30], placental histopathological evaluation was recommended in accordance with American Society of Clinical Oncology (ASCO) and Society for Maternal–Fetal Medicine guidelines [30,31] as placental metastases, although rare, carry implications for maternal staging and neonatal risk [30]. In our patient, placental histopathological examination showed no evidence of metastatic involvement.

### 3.2. Perioperative Management and Anesthetic Strategy

Repeat MRI demonstrated hematoma enlargement and worsening midline shift in the absence of clinical deterioration. Approximately 15 h after ICU admission, neurological status worsened, and the decision was made to proceed with urgent surgical decompression. In this case, continuous intraoperative fetal heart rate (FHR) monitoring was individualized in accordance with ACOG recommendations, considering gestational age (32 + 5 weeks), incomplete antenatal corticosteroid administration, and the institutional capability for emergency cesarean delivery [17,18]. During neurosurgical intervention, FHR monitoring provided real-time information on fetal status and complemented maternal hemodynamic assessment as an indirect indicator of uteroplacental perfusion [18,21].

The observed reduction in fetal heart rate variability during anesthesia was anticipated and consistent with the literature describing the effects of maternal general anesthesia on fetal heart rate patterns [18,21,32]. Baseline fetal heart rate remained normal without pathological patterns, allowing uninterrupted neurosurgical continuation.

Anesthetic management aimed to preserve maternal cerebral perfusion while maintaining uteroplacental blood flow [6,20,32]. Modified rapid sequence induction with head-up position and left lateral tilt was performed to reduce aspiration risk and aortocaval compression, both relevant to advanced gestation [13,20,32]. Remifentanil was administered 60 s before laryngoscopy to attenuate the sympathetic response to intubation [33]. No premedication was administered because of the emergency setting and the patient’s reduced level of consciousness. Tracheal intubation was performed using video laryngoscopy and a tracheal tube with stylet to maximize first-pass success, with backup airway equipment immediately available and predefined team roles established. Total intravenous anesthesia with propofol and remifentanil was selected to optimize intracranial pressure control and brain relaxation, maintain hemodynamic stability, and facilitate rapid postoperative neurological assessment [34]. Anesthetic depth was titrated integrating maternal hemodynamic parameters (heart rate, blood pressure) together with fetal heart rate patterns. Propofol and remifentanil doses were adjusted in real time to avoid maternal awareness while minimizing excessive fetal central nervous system depression from anesthetic agents. Intraoperative invasive neuromonitoring, including evoked potential monitoring, was not performed because of the emergency nature of the procedure.

Hemodynamic management was individualized to optimize both cerebral perfusion pressure and uteroplacental flow. Systolic blood pressure was maintained at 130–140 mmHg, consistent with AHA/ASA 2022, Code ICH consensus, and 2025 AHA/ACC guidelines, avoiding SBP < 130 mmHg which has been associated with harm in acute intracerebral hemorrhage [10,15,35]. In the absence of pregnancy-specific intraoperative MAP targets for neurosurgical procedures, a MAP ≥ 70 mmHg was selected as a pragmatic threshold to balance the risk of maternal hypotension—which may compromise uteroplacental perfusion [32,36]—with the need to avoid excessive hypertension that could worsen cerebral edema or hemorrhage.

Ventilation was titrated to maintain PaCO_2_ at 35–38 mmHg. This range was chosen to avoid hypercapnia-induced ICP elevation while preventing excessive hypocapnia, which causes cerebral vasoconstriction with risk of ischemia and reduces uteroplacental perfusion and fetal oxygenation [20,37,38].

Osmotic therapy was not administered in the absence of clinical signs of transtentorial herniation or documented elevated ICP, in accordance with AHA/ASA 2022 guidelines, which recommend hyperosmolar therapy only for transient reduction in ICP rather than prophylactic use [10]. Moreover, mannitol-induced osmotic diuresis may cause or worsen maternal dehydration and hypovolemia, which could potentially compromise uteroplacental perfusion.

No prophylactic antiseizure medication was administered, consistent with current guidelines [10,39] which do not recommend routine prophylactic antiseizure drugs after ICH. The patient had no clinical seizures preoperatively or intraoperatively.

We did not administer additional corticosteroids for cerebral edema. At presentation, the underlying cause of hemorrhage was unknown, and we followed current guidelines for spontaneous intracerebral hemorrhage, which recommend against corticosteroid use [10]. The patient had received betamethasone (12 mg IM) for fetal lung maturation prior to surgery. Although dexamethasone is recommended for symptomatic brain metastases [39], the diagnosis was confirmed only after histopathological examination on postoperative day 7. By that time, surgical decompression had already relieved the mass effect, making additional corticosteroid therapy unnecessary.

### 3.3. Multidisciplinary Coordination and Role Allocation

The absence of unified protocols for maternal–fetal neurosurgical emergencies represents a significant limitation in time-critical scenarios and relies on institution-specific planning [2,3,5,14]. Prior to surgery, a multidisciplinary team was assembled including neurosurgery, anesthesiology, obstetrics and neonatology, with clearly assigned roles and predefined intervention thresholds for both maternal deterioration and fetal compromise, consistent with recommendations emphasizing that effective team coordination and communication is essential in maternal–fetal emergencies [2,3,14]. Detailed responsibilities for each team member are presented in Table 2.

### 3.4. Operational Readiness for Emergency Delivery

Emergency cesarean delivery was not a theoretical contingency: the obstetric and neonatal teams remained present throughout the procedure, with a sterile field and instruments immediately available and predefined intervention criteria, ensuring the ability to proceed with delivery within minutes. This arrangement allowed uninterrupted neurosurgery while preserving immediate response capability for both patients.

The stepwise multidisciplinary protocol applied to guide clinical management throughout the case is summarized in Table 3.

### 3.5. Strengths and Limitations

This report offers a detailed account of the clinical reasoning behind each decision in a single, specific case. The organizational principles described—predefined role allocation, shared intervention thresholds, and structured communication—do not require advanced technology. While potentially informative for other teams managing similar situations, their applicability across different clinical contexts remains uncertain. Cross-border collaboration between tertiary centers supported continuity of care and access to complementary expertise but should be regarded as a contextual aspect of management rather than a proven determinant of outcome.

The limitations are those inherent to any single case report. Although favorable maternal and fetal outcomes were observed, causality cannot be attributed to any specific component of management and may simply reflect fortunate circumstances, including lesion characteristics, gestational age, and the expertise available at the time. This case represents a highly specific clinical scenario. Hemorrhagic melanoma brain metastasis during pregnancy is rare [28,29] and differs substantially from the broader population of pregnancy-associated intracerebral hemorrhage, which is more commonly related to hypertensive disorders, vascular malformations, or coagulopathies. Consequently, the management approach described was individualized to the patient’s specific etiology, gestational age, hemodynamic status, and institutional setting, and should not be considered directly transferable to other clinical contexts. In addition, the favorable maternal and fetal outcome observed in this case may have been influenced by case-specific factors, including hematoma characteristics, preserved neurological status at the time of surgical intervention, and the availability of tertiary multidisciplinary expertise, rather than any individual organizational or therapeutic strategy. Finally, long-term maternal neurological and oncological outcomes were not available, as subsequent care was provided at the referral center, representing an additional limitation of this report.

## 4. Conclusions

This case report describes the perioperative management of a single patient who underwent emergency craniotomy during late pregnancy for hemorrhagic melanoma brain metastasis. It documents one institutional experience with this rare clinical scenario and illustrates the implementation of a structured multidisciplinary approach. However, it does not establish safety standards, define a reproducible protocol, or support broader conclusions regarding the management of neurosurgical emergencies during pregnancy.

Favorable maternal and fetal outcomes occurred in the context of structured multidisciplinary coordination, continuous intraoperative fetal monitoring, and preparedness for emergency cesarean delivery; however, the contribution of any individual component to outcome cannot be determined from a single clinical experience. Nonetheless, some observations from this case may provide considerations relevant to similar clinical situations. A systematic differential diagnosis and time-sensitive emergency department evaluation established the initial management framework. Close clinical and radiological monitoring, together with structured multidisciplinary reassessment, supported escalation to surgical decompression when neurological deterioration and hematoma expansion became apparent. Intraoperative fetal heart rate monitoring, individualized according to ACOG recommendations, provided assessment of fetal status during general anesthesia, facilitating interpretation of transient fetal heart rate changes in the context of expected anesthesia-related physiological effects. The continuous presence of obstetric and neonatal teams ensured preparedness for emergency cesarean delivery. Histopathological examination of the evacuated hematoma confirmed hemorrhagic melanoma brain metastasis, substantially influencing subsequent oncological and obstetric management. This finding further limits the applicability of this experience to the broader population of pregnant patients with intracerebral hemorrhage of other etiologies. Additional evidence is required to better understand which organizational and clinical approaches may be useful in different contexts.

## 5. Lessons from the Case

The following reflect the clinical reasoning applied in this specific case.

- Although uncommon, neoplastic lesions should be considered among the differential diagnoses of intracerebral hemorrhage during pregnancy. In the present case, the diagnosis of hemorrhagic melanoma brain metastasis was established only after histopathological examination of the evacuated hematoma.
- Hemodynamic management required balancing competing maternal cerebral and uteroplacental physiological demands. Blood pressure targets were selected pragmatically to reduce the risk of hematoma expansion while preserving uteroplacental perfusion; whether these thresholds were optimal remains uncertain.
- Readiness for emergency cesarean delivery extended beyond team availability. In this case, obstetric and neonatal teams were present throughout the procedure with a sterile field immediately available, enabling potential delivery within minutes if maternal or fetal deterioration had occurred.
- Predefined role allocation, shared intervention thresholds, and structured communication supported decision-making in this specific context. It is unclear whether similar approaches would be feasible in other institutions.

## Figures and Tables

**Figure 1:**
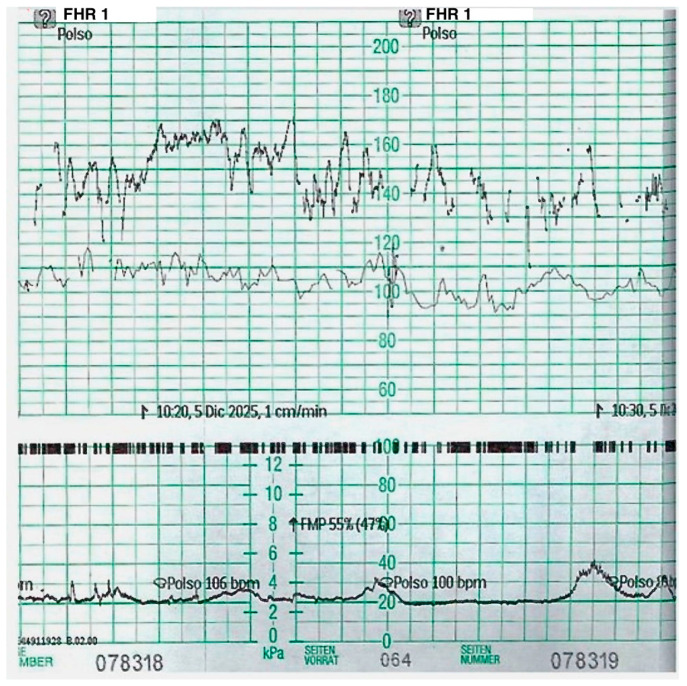
CTG monitoring during anesthesia induction. The CTG initially demonstrated a baseline fetal heart rate within the normal range (110–160 bpm), with normal variability and reactivity; a progressive reduction in FHR variability and reactivity is observed. No uterine contractions are present.

**Figure 2:**
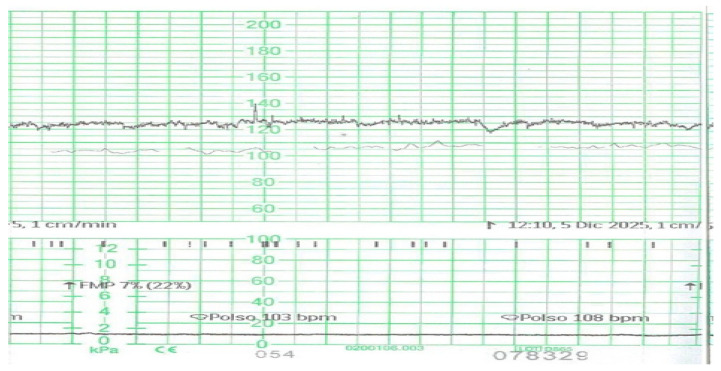
Intraoperative CTG monitoring. The baseline FHR remains within the normal range (approximately 135 bpm), with absent variability and reactivity, consistent with the sedative effects of maternal anesthesia, yet no decelerations or other suspicious patterns are noted. Uterine activity remains absent.

**Figure 3:**
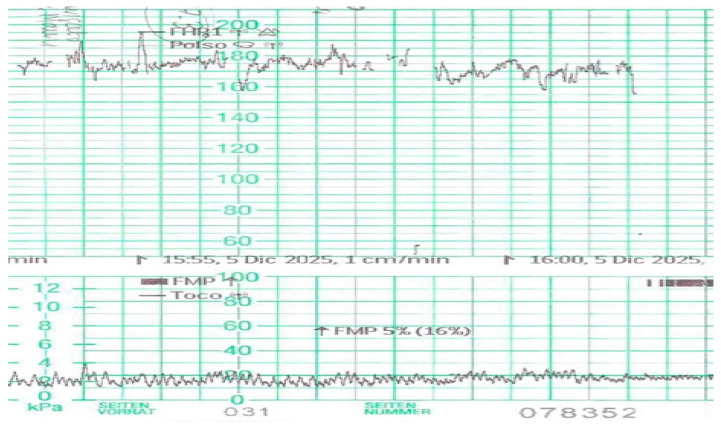
CTG monitoring upon recovery from anesthesia. Fetal tachycardia is evident, with a baseline FHR of 170 bpm, partially attributable to increased fetal movements. A gradual restoration of FHR variability and reactivity is observed, while uterine contractile activity remains absent.

**Figure 4:**
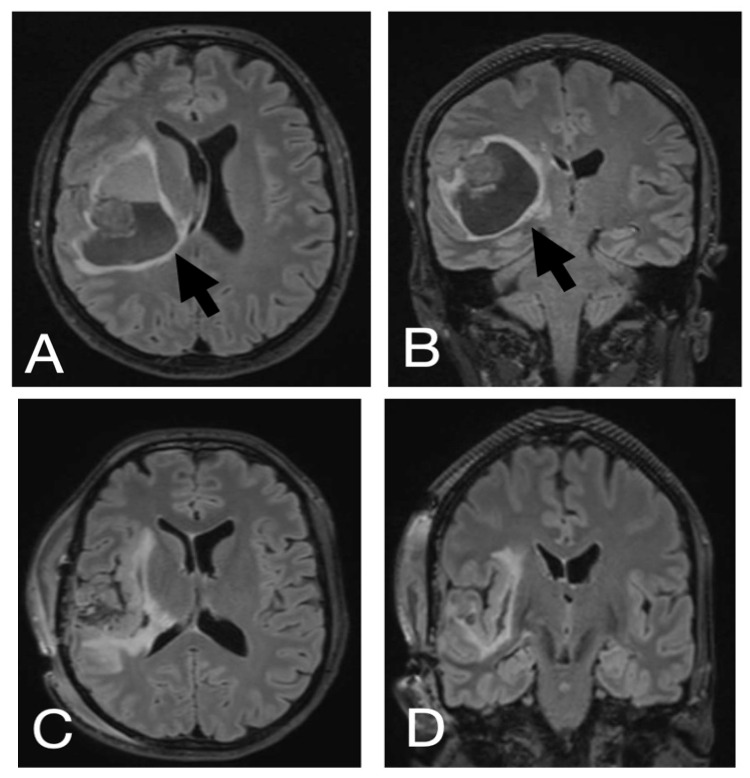
Preoperative (**A**,**B**) and postoperative (**C**,**D**) MRI FLAIR sequences demonstrating complete removal of the hematoma and resolution of the associated mass effect. In images (**A**,**B**), a suspected lesion is clearly visible (arrow), later confirmed as melanoma on histopathological analysis.

**Table 1:** Chronological timeline of clinical presentation, diagnostic work-up, multidisciplinary management, surgical treatment, and follow-up.

Time/Day	Clinical Event
Day 0–11:30	Last known well, subsequently developed mild left-hand weakness.
Day 0–12:30	Sudden worsening of left-hand weakness with rapid progression to left-sided hemiparesis.
Day 0–13:15	Emergency department arrival (105 min from symptom onset); initial triage, neurological assessment (NIHSS 18).
Day 0–13:30	Non-contrast head CT: Heterogeneous right capsulo-insular hemorrhage (50 mm × 40 mm × 35 mm) with associated mass effect and a 4 mm midline shift in the septum pellucidum. No signs of herniation.
Day 0–13:35	Initial blood pressure assessment (115/72 mmHg) and serial monitoring, targeting systolic blood pressure < 140 mmHg per current guidelines [10,15].
Day 0–14:00	Brain MRI and MR angiography: Right insular intra-axial hematoma (50 mm × 38 mm × 35 mm, estimated volume 33 mL) with mass effect and a 4 mm shift in the septum pellucidum. An underlying lesion could not be ruled out.
Day 0–15:00	Obstetric evaluation: Reassuring fetal status, normal cardiotocography (CTG), no signs of labor.
Day 0–15:30	Neurosurgical and multidisciplinary consultation (neurology, neurosurgery, obstetrics, anesthesia); decision for initial conservative management given maternal neurological stability and fetal prematurity.
Day 0–17:00	Admission to intensive care unit (ICU) for close neurological and maternal–fetal monitoring.
Day 0–1	Conservative management, twice daily cardiotocography (CTG).
Day 0–18:00	Antenatal corticosteroids were initiated with betamethasone 12 mg IM (first of two doses) for fetal lung maturation, according to American College of Obstetricians and Gynecologists (ACOG) guidelines [16].
Day 0–20:30	Repeat MRI demonstrated hematoma expansion (60 mm × 44 mm × 46 mm, estimated volume 61 mL), progression of midline shift to 5 mm, with increased mass effect and effacement of the lateral ventricle. No radiological signs of herniation.
Day 1–8:00	Acute neurological deterioration with worsening deficit (NIHSS 19).
Day 1–9:00	Urgent multidisciplinary reassessment with decision for emergency craniotomy due to neurological deterioration and radiological progression. Administration of preoperative tocolysis (intravenous hexoprenaline infusion 6.4 µg/h).
Day 1 (Intraoperative) Surgery start time 11:50	Right temporal craniotomy, hematoma evacuation, lesion exploration, continuous fetal monitoring.
Day 1 (Postoperative day 0)–18:00	Second dose of antenatal betamethasone (12 mg IM), completing the course.
Day 1–3 (Postoperative day 0–2)	ICU monitoring with progressive neurological improvement.
Day 3 (Postoperative day 2)	Transfer to neurosurgery ward (NIHSS 14).
Day 7	Histopathological diagnosis: Hemorrhagic metastasis of malignant melanoma (immunohistochemistry with mutated BRAF V600E).
Day 13	Transfer to tertiary referral center for ongoing maternal–fetal and oncological management (NIHSS 5).
Gestational Week 36 + 0	Elective cesarean delivery performed 3 weeks after craniotomy. Birth weight and Apgar scores unavailable (delivery at an external hospital abroad, corresponding medical records not accessible).

**Table 2:** Speciality-specific roles and responsibilities in multidisciplinary preoperative management.

Specialty	Role/Intervention
Neurosurgery	Right temporal craniotomy, hematoma evacuation, lesion exploration, tissue sampling for histopathology.
Anesthesia	Balance maternal neuroprotection (ICP control, cerebral perfusion) with uteroplacental perfusion; total intravenous anesthesia (TIVA) with target-controlled infusion (TCI).
Obstetrics	Continuous intraoperative fetal monitoring; readiness for emergency cesarean, preoperative tocolysis administration (intravenous hexoprenaline infusion (6.4 µg/h) until postoperative day 1).
Neonatology	Intraoperative presence, immediate neonatal resuscitation capability if emergency delivery required.

**Table 3:** Multidisciplinary decision-making process outlining a stepwise approach integrating maternal and fetal parameters to guide real-time clinical decisions in a pregnant patient with intracerebral hemorrhage.

Step	Domain	Key Elements	Decision/Outcome
1	Initial assessment	Neurological status (GCS, NIHSS); Neuroimaging; Fetal condition (CTG, gestational age)	Define severity and urgency
2	Multidisciplinary evaluation	Neurosurgery; Anesthesia; Obstetrics; Neonatology	Shared decision-making
3	Surgical indication	Hematoma expansion; Neurological deterioration; Mass effect	**Yes** → proceed to surgery/ **No** → conservative management
4	Shared planning	Role allocation; Emergency cesarean readiness; Continuous fetal monitoring	Coordinated perioperative strategy
5	Anesthesia	Neuroprotection; Hemodynamic stability; Maintenance of cerebral and uteroplacental perfusion	Physiological balance (maternal–fetal)
6	Intraoperative phase	Integrated maternal–fetal monitoring	Real-time assessment
7	Maternal–fetal stability	Maternal hemodynamic + fetal heart rate	**Stable** → continue surgery/ **Unstable** → further evaluation
8	Fetal status (if instability)	CTG abnormalities (bradycardia, decelerations, etc.)	**Distress** → emergency cesarean/**No distress** → optimize management
9	Postoperative care	ICU monitoring; Neurological and fetal reassessment	Stabilization phase

## Data Availability

This study is based on a single-patient case report; no structured dataset was generated or analyzed. All relevant clinical information is presented within the manuscript. Additional patient data (clinical records, operative and anesthetic documentation) cannot be shared publicly due to privacy and confidentiality restrictions.
